# Supplementary material for: DARUMA: a gateway to fast and easy prediction of intrinsically disordered regions
Source: PeerJ Comput Sci. 2025 Nov 14;11:e3343. doi: 10.7717/peerj-cs.3343 (PMC13293392; doi:10.7717/peerj-cs.3343)
Supplement: Supplemental Information 1 [file peerj-cs-11-3343-s001.pdf]

Supplemental Articles S1: Optimization of hyperparameters for DARUMA.

The optimization of DARUMA's hyperparameters was performed in two steps. First, six combinations of hyperparameters (listed in Table S2B) for the PU were selected through a grid search against the candidate hyperparameters detailed in Table S2A. In this phase, the original L × 553-dimensional vector served as the input data for the PU, and the top six combinations with the highest MCC were identified. Subsequently, the structure of the PU and the hyperparameters of the FEU were fixed by conducting another grid search against the candidate hyperparameters outlined in Table S2B and Table S2C, respectively. Consequently, the size of the window, the number of affine layers, and the number of nodes in the affine layers of the PU were set to 121, 2, and 128, respectively. For the FEU, the number and size of the filter in the 1D Convolutional layers were determined to be 3 and 9, respectively.
