# Supplementary figures and images for: DARUMA: a gateway to fast and easy prediction of intrinsically disordered regions
Source: PeerJ Comput Sci. 2025 Nov 14;11:e3343. doi: 10.7717/peerj-cs.3343 (PMC13293392; doi:10.7717/peerj-cs.3343)

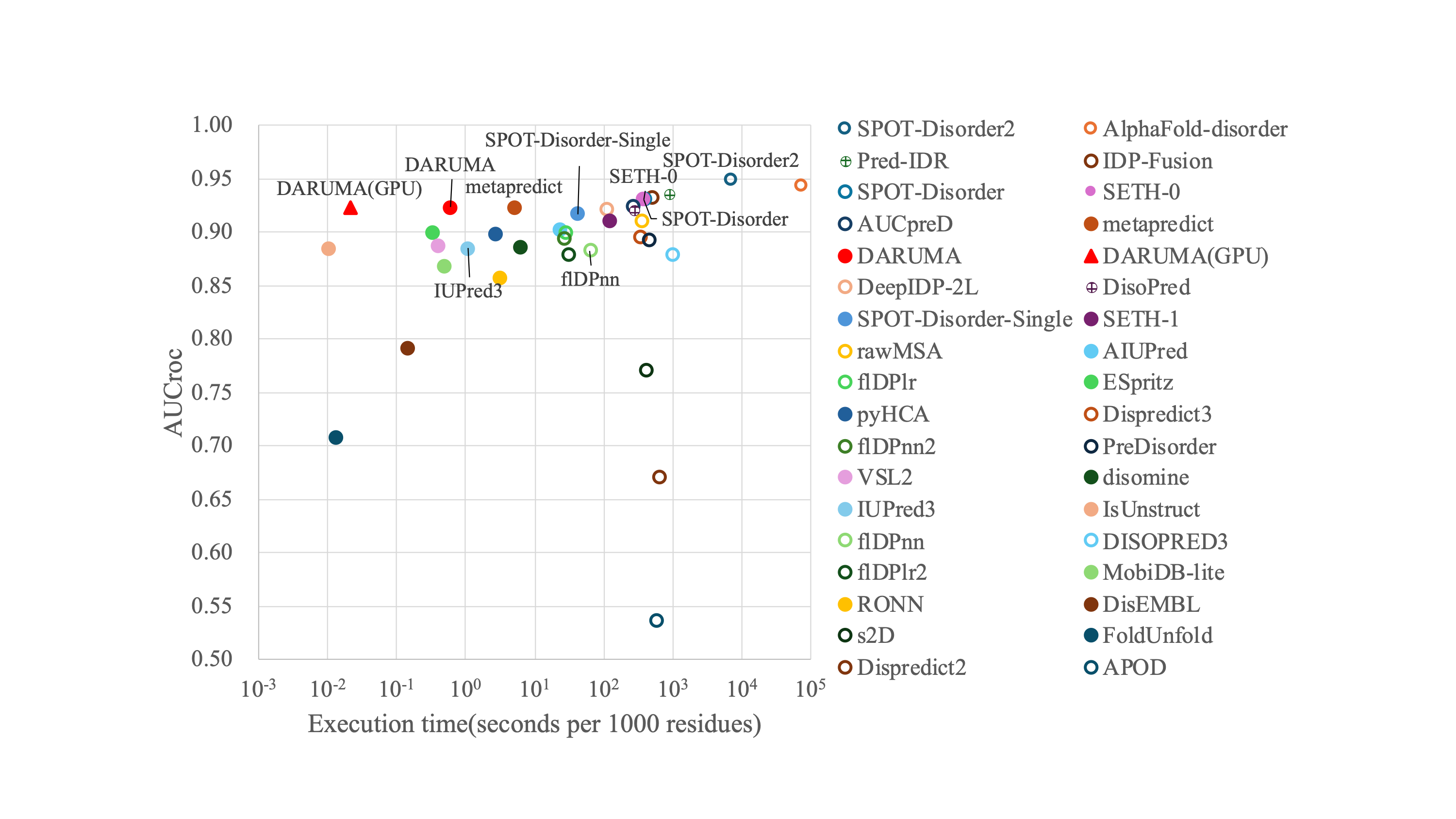

Supplement: Supplemental Information 2 — Execution time and AUCroc for each predictor are plotted on the horizontal and vertical axes. Filled and open circles indicate the Profile-free predictors and Profile-driven predictors, respectively. The Pred-IDR and DisoPred are plotted as a lattice of circles because the details of both predictors have not been published. A red triangle indicates DARUMA executed on the GPU. Please note that the horizontal axis is presented on a logarithm scale. [file peerj-cs-11-3343-s002.png]

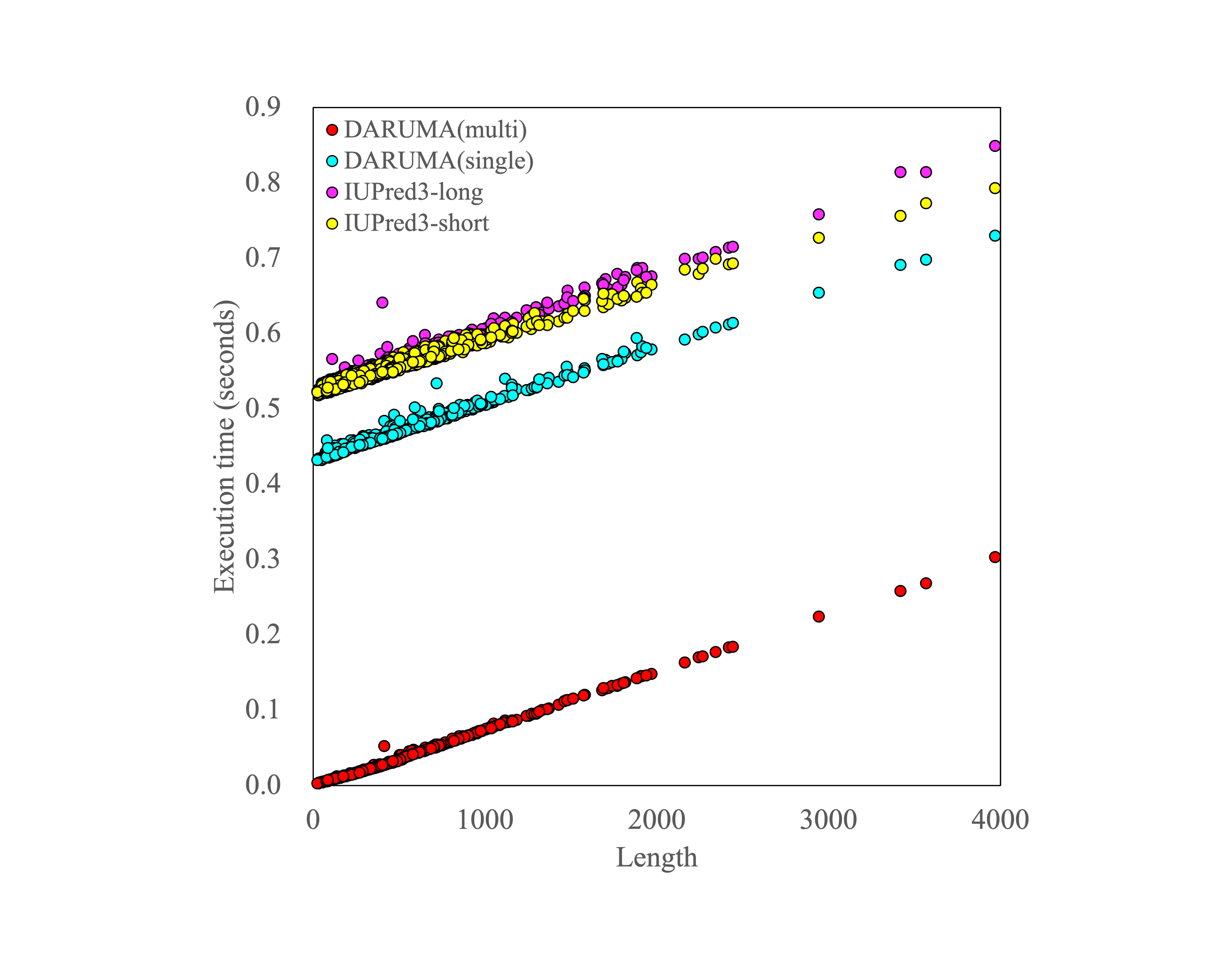

Supplement: Supplemental Information 3 — The circles represent the execution times of proteins for DARUMA(multi) (red circles), DARUMA(single) (cyan circles), IUPred3-long (magenta circles), and IUPred3-short (yellow circles), respectively. [file peerj-cs-11-3343-s003.png]
