# Supplementary material for: DARUMA: a gateway to fast and easy prediction of intrinsically disordered regions
Source: PeerJ Comput Sci. 2025 Nov 14;11:e3343. doi: 10.7717/peerj-cs.3343 (PMC13293392; doi:10.7717/peerj-cs.3343)
Supplement: Supplemental Information 4 [file peerj-cs-11-3343-s004.docx]

**Supplemental Table S1: Dataset details.**

|  | dataset name | protein | order | disorder | undefined |
| --- | --- | --- | --- | --- | --- |
| Training data | DM4229 | 3,731 | 819,404 | 86,606 | 0 |
|  | IDEAL | 555 | 89,614 | 23,847 | 277,644 |
| Validation data1 | DM4229 | 414 | 97,348 | 10,229 | 0 |
|  | IDEAL | 62 | 10,099 | 3,482 | 33,232 |
| Validation data2 | SL | 239 | 35,773 | 25,876 | 63,341 |
| Test data1(CAID1) | DisProt646 | 646 | 281,991 | 54,604 | 0 |
|  | DisProt-PDB646 |  | 122,466 |  | 159,525 |
| Test data2(CAID2) | Disorder-NOX | 210 | 129,487 | 31,315 | 0 |
|  | Disorder-PDB | 348 | 93,805 | 37,072 | 156,143 |
