# Supplementary material for: DARUMA: a gateway to fast and easy prediction of intrinsically disordered regions
Source: PeerJ Comput Sci. 2025 Nov 14;11:e3343. doi: 10.7717/peerj-cs.3343 (PMC13293392; doi:10.7717/peerj-cs.3343)
Supplement: Supplemental Information 5 [file peerj-cs-11-3343-s005.docx]

**Supplemental Table S2: List of candidate hyperparameters for grid search.**

**A** Candidate hyperparameters for the PU.

|  | Candidate hyperparameters |
| --- | --- |
| The size of the window | {61, 81, 101, 121, 141} |
| The number of Affine layers | {1, 2} |
| The number of nodes in Affine layers | {64, 128, 256, 512, 1024} |

**B** Candidate combination of hyperparameters for the PU.

|  | Combinations  (window size, #layers, #nodes) |
| --- | --- |
| Candidate1 | (101, 1, 128) |
| Candidate2 | (101, 1, 256) |
| Candidate3 | (101, 1, 512) |
| Candidate4 | (121, 2, 128) |
| Candidate5 | (121, 2, 256) |
| Candidate6 | (121, 2, 512) |

**C** Candidate hyperparameters for the FEU.

|  | Candidate hyperparameters |
| --- | --- |
| The size of the window | {1, 2, 3, 4, 5, 6, 7, 8} |
| The number of nodes in Affine layers | {3, 5, 7, 9} |
