# Supplementary material for: DARUMA: a gateway to fast and easy prediction of intrinsically disordered regions
Source: PeerJ Comput Sci. 2025 Nov 14;11:e3343. doi: 10.7717/peerj-cs.3343 (PMC13293392; doi:10.7717/peerj-cs.3343)
Supplement: Supplemental Information 6 — The order of predictors follows based on AUCroc. The MCC and AUCroc for all predictors excluding DARUMA and DARUMA (GPU) were calculated based on the prediction results provided in https://caid.idpcentral.org/assets/sections/challenge/static/predictions/2/predictions.zip . The execution times for all predictors excluding DARUMA and DARUMA (GPU) were obtained from the results reported on https://caid.idpcentral.org/challenge/results . We executed DARUMA on the Intel Xeon E5-2697 with RAM limited to 47 GB to fairly compare the execution time. We run DARUMA(GPU) on the Intel(R) Xeon(R) Gold 5222 CPU @ 3.80GHz with GPU of RTX2080ti. The ’D’, ’F’, or ’-’ in the Profile column indicates the predictor group, Profile-driven, Profile-free, or not published, respectively. [file peerj-cs-11-3343-s006.docx]

**Supplemental Table S3: Two accuracy metrics and execution time for all predictors on disorder_pdb.**

| Predictor | MCC | AUCroc | Execution time(s/1000res) | Profile |
| --- | --- | --- | --- | --- |
| SPOT-Disorder2 | 0.794 | 0.949 | 6911.79 | D |
| AlphaFold-disorder | 0.781 | 0.944 | 71690.67 | D |
| Pred-IDR | 0.706 | 0.934 | 893.31 | - |
| IDP-Fusion | 0.755 | 0.933 | 503.28 | D |
| SPOT-Disorder | 0.755 | 0.931 | 380.54 | D |
| SETH-0 | 0.765 | 0.930 | 372.18 | F |
| AUCpreD | 0.715 | 0.924 | 263.56 | D |
| metapredict | 0.757 | 0.923 | 5.07 | F |
| **DARUMA** | **0.737** | **0.923** | **0.60** | **F** |
| **DARUMA(GPU)** | **0.737** | **0.923** | **0.021** | **F** |
| DeepIDP-2L | 0.711 | 0.922 | 107.56 | D |
| DisoPred | 0.693 | 0.919 | 283.71 | - |
| SPOT-Disorder-Single | 0.667 | 0.917 | 41.49 | F |
| SETH-1 | 0.711 | 0.911 | 120.42 | F |
| rawMSA | 0.639 | 0.910 | 347.39 | D |
| AIUPred | 0.640 | 0.903 | 22.35 | F |
| flDPlr | 0.416 | 0.900 | 27.60 | D |
| ESpritz | 0.475 | 0.899 | 0.33 | F |
| pyHCA | 0.664 | 0.898 | 2.67 | F |
| Dispredict3 | 0.470 | 0.895 | 338.83 | D |
| flDPnn2 | 0.434 | 0.894 | 26.61 | D |
| PreDisorder | 0.606 | 0.893 | 439.53 | F |
| VSL2 | 0.573 | 0.887 | 0.40 | D |
| disomine | 0.496 | 0.886 | 6.015 | F |
| IUPred3 | 0.652 | 0.885 | 1.071 | F |
| IsUnstruct | 0.620 | 0.885 | 0.010 | F |
| flDPnn | 0.540 | 0.883 | 63.68 | D |
| DISOPRED3 | 0.634 | 0.879 | 961.75 | D |
| flDPlr2 | 0.554 | 0.879 | 29.93 | D |
| MobiDB-lite | 0.558 | 0.868 | 0.48 | F |
| RONN | 0.550 | 0.857 | 3.138 | F |
| DisEMBL | 0.428 | 0.792 | 0.141 | F |
| s2D | 0.332 | 0.771 | 417.219 | D |
| FoldUnfold | 0.525 | 0.708 | 0.013 | F |
| Dispredict2 | 0.250 | 0.671 | 621.007 | D |
| APOD | 0.179 | 0.536 | 566.531 | D |

The order of predictors follows based on AUCroc. The MCC and AUCroc for all predictors excluding DARUMA and DARUMA (GPU) were calculated based on the prediction results provided in <https://caid.idpcentral.org/assets/sections/challenge/static/predictions/2/predictions.zip>. The execution times for all predictors excluding DARUMA and DARUMA (GPU) were obtained from the results reported on <https://caid.idpcentral.org/challenge/results>. We executed DARUMA on the Intel Xeon E5-2697 with RAM limited to 47 GB to fairly compare the execution time. We run DARUMA(GPU) on the Intel(R) Xeon(R) Gold 5222 CPU @ 3.80GHz with GPU of RTX2080ti. The 'D', 'F', or '-' in the Profile column indicates the predictor group, Profile-driven, Profile-free, or not published, respectively.
